# Phosphorylation of pyruvate kinase M2 and lactate dehydrogenase A by fibroblast growth factor receptor 1 in benign and malignant thyroid tissue

**DOI:** 10.1186/s12885-015-1135-y

**Published:** 2015-03-18

**Authors:** Paul Kachel, Bogusz Trojanowicz, Carsten Sekulla, Hanna Prenzel, Henning Dralle, Cuong Hoang-Vu

**Affiliations:** 1 Department of General, Visceral and Vascular Surgery, Faculty of Medicine, Martin-Luther-University of Halle-Wittenberg, Halle/Saale, Germany; 2 Department of Internal Medicine II, Faculty of Medicine, Martin-Luther-University of Halle-Wittenberg, Halle/Saale, Germany

**Keywords:** Pyruvate Kinase M2, Lactate dehydrogenase A, Fibroblast growth factor receptor 1, Warburg effect, Thyroid, Tumour marker

## Abstract

**Background:**

Lactate dehydrogenase A (LDHA) and Pyruvate Kinase M2 (PKM2) are important enzymes of glycolysis. Both of them can be phosphorylated and therefore regulated by Fibroblast growth factor receptor 1 (FGFR1). While phosphorylation of LDHA at tyrosine10 leads to tetramerization and activation, phosphorylation of PKM2 at tyrosine105 promotes dimerization and inactivation. Dimeric PKM2 is found in the nucleus and regulates gene transcription. Up-regulation and phosphorylation of LDHA and PKM2 contribute to faster proliferation under hypoxic conditions and promote the Warburg effect.

**Methods:**

Using western blot and SYBR Green Real time PCR we investigated 77 thyroid tissues including 19 goiter tissues, 11 follicular adenomas, 16 follicular carcinomas, 15 papillary thyroid carcinomas, and 16 undifferentiated thyroid carcinomas for total expression of PKM2, LDHA and FGFR1. Additionally, phosphorylation status of PKM2 and LDHA was analysed. Inhibition of FGFR was performed on FTC133 cells with SU-5402 and Dovitinib.

**Results:**

All examined thyroid cancer subtypes overexpressed PKM2 as compared to goiter. LDHA was overexpressed in follicular and papillary thyroid cancer as compared to goiter. Elevated phosphorylation of LDHA and PKM2 was detectable in all analysed cancer subtypes. The highest relative phosphorylation levels of PKM2 and LDHA compared to overall expression were found in undifferentiated thyroid cancer. Inhibition of FGFR led to significantly decreased phosphorylation levels of PKM2 and LDHA.

**Conclusions:**

Our data shows that overexpression and increased phosphorylation of PKM2 and LHDA is a common finding in thyroid malignancies. Phospho-PKM2 and Phospho-LDHA could be valuable tumour markers for thyroglobulin negative thyroid cancer.

**Electronic supplementary material:**

The online version of this article (doi:10.1186/s12885-015-1135-y) contains supplementary material, which is available to authorized users.

## Background

The Warburg effect describes a general feature of cancer cells to show elevated glucose uptake and lactate production even in the presence of oxygen [1]. Warburg proposed an impaired glucose oxidation, which leads to extensive excretion of lactate under normoxia [2]. However, recent data showed that the Warburg effect is common not only in cancer cells but also in induced pluripotent stem cells [3] and in proliferating T cells [4]. These findings raise many questions related to cancer specific alterations in glycolysis and their possible use as prognostic or therapeutic targets.

Pyruvate Kinase (PK), catalysing the step from phosphoenolpyruvate to pyruvate, is a key enzyme of glycolysis. Furthermore it is an important regulator of the Warburg effect. In thyroid tissue there are two isoenzymes: pyruvate kinase M1 (PKM1) and pyruvate kinase M2 (PKM2), which result from alternative splicing of the PKM gene [5]. Bluemlein et al. showed that PKM2 is the dominant isoenzyme in all examined benign and malignant thyroid tissues [6] (Additional file 1). Higher levels of PKM2 in tumour tissues contribute to growth advantage and faster progression in xenograft models as compared to cancer cells expressing PKM1 [7]. However, elevated levels of inactivated dimeric PKM2 are found in cancer cells [8]. This inactivation may be promoted by different mechanisms [9,10] and suggests that PKM2 may possess other, non-glycolytic functions such as regulation of transcription. In addition to these effects involvement of other proteins, which may dramatically affect the function of PKM2, has been reported [10,11]. It has been demonstrated that phosphorylation of tyrosine 105 of PMK2 by fibroblast growth factor receptor 1 (FGFR1) prevents tetramerization and inactivates PKM2. As a consequence this leads to faster proliferation under hypoxic conditions and increased tumour growth in xenograft models [12]. The enzymatically inactivated dimeric form of PKM2 can be translocated to the nucleus and may act as a protein kinase regulating gene transcription implicated in tumour growth [13-16]. Inactivation of PKM2 leads to accumulation of upstream glycolytic metabolites and activation of the pentose-phosphate pathway, hexosamine-pathway and serine biosynthesis. This results in increased availability of metabolites for redox control and nucleotide biosynthesis [11,17]. With regard to clinical employment as a tumourmarker, a great diagnostic and prognostic potential of PKM2 has been demonstrated for several malignancies including oesophagus, pancreas or colorectal cancer [18,19]. However, data concerning PKM2 in thyroid cancer is still lacking.

Lactate dehydrogenase (LDH) catalyses the conversion from pyruvate to lactate. Active LDH consists of four monomers. The two different monomers lactate dehydrogenase A (LDHA) and lactate dehydrogenase B (LDHB) are expressed in an organ depending manner. While LDHA preferentially turns pyruvate into lactate, LDHB works the opposite way [20].

LDHA is up-regulated in a wide range of tumour tissues including lung, breast, endometrium, urinary bladder, testicular germ cell and large intestine cancers [20,21]. Down-regulation or inhibition of LDHA resulted in decreased ATP levels, reduced mitochondrial membrane potential and an increase in oxidative stress that is linked to cell death. Furthermore, decreased levels of LDHA were related to inhibition of tumour xenograft maintenance and progression [22,23]. Investigations on thyroid tissues showed a decreased LDHA/LDHB ratio in thyroid oncocytoma and follicular tumours compared to normal thyroid tissue which was altered by estrogen related receptor alpha. Those tumours were more dependent on oxidative phosphorylation [24]. In contrast to PKM2, FGFR1-mediated phosphorylation of LDHA at tyrosine 10 (y10) promotes tetramerization and turns LDHA into the active enzyme [25].

Fibroblast growth factor receptors (FGFR) play an important role in many human malignancies, such as bladder or breast cancer [26,27]. They are involved in the regulation of cellular proliferation, differentiation, migration and cell survival [28]. With regard to thyroid tissue, the most studied receptor of this family, FGFR1, is overexpressed in differentiated thyroid cancer and in thyroid hyperplasia [29,30].

So far, FGFR1-mediated phosphorylation effects on LDHA and PKM2 have only been demonstrated in cell culture experiments without any relevance to human cancer tissue, especially thyroid carcinoma.

In this study we investigated the expression of PKM2, LDHA and FGFR1 in thyroid benign and malignant tissues by employment of qPCR and western blot. Additionally, a possible impact of FGFR1-mediated phosphorylation of PKM2 and LDHA on thyroid malignancy was evaluated.

## Methods

### Tissue

A total of 77 thyroid tissue samples, including 19 goiter tissues, 11 follicular adenomas (FA), 16 follicular carcinomas (FTC), 15 papillary thyroid carcinomas (PTC) and 16 undifferentiated thyroid carcinomas (UTC) were collected from patients at the surgical institute of the Martin Luther University Halle Wittenberg. The study was approved by the ethical committee of the medical department of the Martin Luther University Halle Wittenberg. All patients gave written consent.

### Protein isolation and western blot analysis

Frozen tissue samples were collected directly after surgery and stored in liquid nitrogen. Tissue specimens were divided and sections where prepared and stained with hematoxylin and eosin to confirm the pathological diagnosis. After confirmation frozen tissue specimens were homogenised and further divided for RNA- or protein extraction. For protein extraction the homogenate was lysed using a protein lysis buffer (7 M urea, 2 M thiourea, 4% Chaps, 40 mM DTT). Protein concentration was measured by using the Bradford assay method. Proteins were separated by SDS-PAGE blotted on PVDF membrane and blocked with 5% BSA for one hour. They were incubated overnight in a 1:1000 dilution of the primary antibody. β-actin was used as normalizing marker. Total lysate from cell line FTC133 was used as positive control. FTC133 is a thyroid cancer cell line, which was obtained from a lymph node metastases from a 42-year-old male with follicular thyroid cancer. As proven in preliminary experiments FTC133 expresses PKM1, PKM2, LDHA and FGFR1 on mRNA and protein levels (Additional file 2). Membranes were first stained with Phospho-PKM2 or Phospho-LDHA antibody, respectively. Subsequently, they were treated with stripping buffer (15 g glycin, 1 g SDS, 10 ml Tween adjusted to pH 2.2 and filled up with H_2_O to 1 l) and tested for remaining signals. They were again incubated overnight with total LDHA or PKM2 antibody, respectively. Protein expression was measured in relation to FTC133 as positive control and then normalized to the expression of ß-actin as a normalizing marker. Differences are expressed in percent to positive control defined as 100%. In order to evaluate the expression and phosphorylation status of PKM2 and LDHA we subjected total protein lysates to western blot with specific antibodies raised against total PKM2 and LDHA, phosphorylated y105 of PKM2 and phosphorylated y10 od LDHA. To evaluate the expression of FGFR1 antibodies against total FGFR1 were used (Table 1).

**Table 1:** **Antibodies**

Antibody	Company
PKM2 Antibody	Cell Signaling Technology, Cambridge, England
Phospho-PKM2 (Tyr105) Antibody	Cell Signaling Technology, Cambridge, England
LDHA (C4B5) Rabbit mAb	Cell Signaling Technology, Cambridge, England
Phospho-LDHA (Tyr10) Antibody #8176	Cell Signaling Technology, Cambridge, England
FGF Receptor 1 (D8E4)	Cell Signaling Technology, Cambridge, England
Monoclonal Anti-ß-Aktin Clone AC15	Sigma Aldrich, Saint Louis, USA
Goat anti Rabbit IgG HRP	Santa Cruz Biotechnology, Santa Cruz, USA
Goat anti Mouse IgG HRP	Santa Cruz Biotechnology, Santa Cruz, USA
PKM1 Rabbit Polyclonal Ab	Signalway Antibody, Maryland, USA

Specific primary and secondary antibodies used for western blot analysis.

### Q-RT-PCR

The transcripts of FGFR1, PKM and LDHA were investigated by employment of quantitative RT-PCR and primer pairs listed in Table 2. 1 μg of total RNA was reversely transcribed by using SuperScript II Kit according to the manufacturer’s instructions (Invitrogen). qPCR was conducted with Rotor Gene SYBR Green PCR Kit (Qiagen) on Rotor Gene Q® Qiagen 2 plax Platform (Qiagen). The cycling conditions were as follows: PCR initial activation step for 5 min. at 95°C, 40 cycles of two step cycling which included denaturation for 5 sec at 95°C and 10 sec of combined annealing and extension at the individual primer temperature between 57°C and 62.5°C. Relative mRNA levels of the transcripts were measured in relation to FTC133 as positive control and then normalized to the expression of the housekeeping genes YWHAZ and GAPDH. Differences are expressed in percent in relation to positive control defined as 100%.

**Table 2:** **Specific primer**

Targetgene	Sense	Antisense
PK-M1/2	5′CTG GGA AGC CTG TCA TCT GT-3′	5′- AGT CCC CTT TGG CTG TTT CT-3′
LDHA	5′-GGC CTC TGC CAT CAG TAT CT-3′	5′-GCC GTG ATA ATG ACC AGC TT-3′
FGFR1	5′-ACA CTG CGC TGG TTG AAA A- 3′	5′-TGG TAT GTG TGG TTG ATG CTC- 3′
yWHAZ (Housekeeping)	5′-AGC AGG CTC AGC GAT ATG AT-3′	5′-TCT CAG CAC CTT CCG TCT TT-3′
GAPDH (Housekeeping)	5′-ACC CAG AAG ACT GTG GAT GG-3′	5′-TTC TAG ACG GCA GGT CAG GT-3′

Specific primers for target genes and housekeeping genes used for SYBR Green Q-RT-PCR.

### Cell culture experiments

8505C and FTC133 cells were grown in DMEM/F12 suppplemented with 10% FCS and 1% PenStrep and incubated at 37°C, 5% CO_2_. For B-CPAP RPMI 1640 medium was used. FGFR1 inhibition experiments were performed on FTC133 cells by employment of Receptor Tyrosine Kinase Inhibitors TKI-258 (Dovitinib, Biomol) and SU-5402 (Sigma-Aldrich). Inhibition was conducted over 4 h with the indicated inhibitor concentrations. Control cells received corresponding concentrations of DMSO.

### Statistical analysis

Statistical analyses were performed with IBM SPSS (version 20) software by employment of a priori test. In case of p < 0.05 a Kruskal Wallice and Mann Whitney U test for subgroup analysis was performed. For correlation analysis the Pearson coefficient was used. Boxplots were performed according to Tukey’s definition.

## Results

### Expression and phosphorylation of y105 of pyruvate kinase M2 (PKM2)

**Figure 1:**
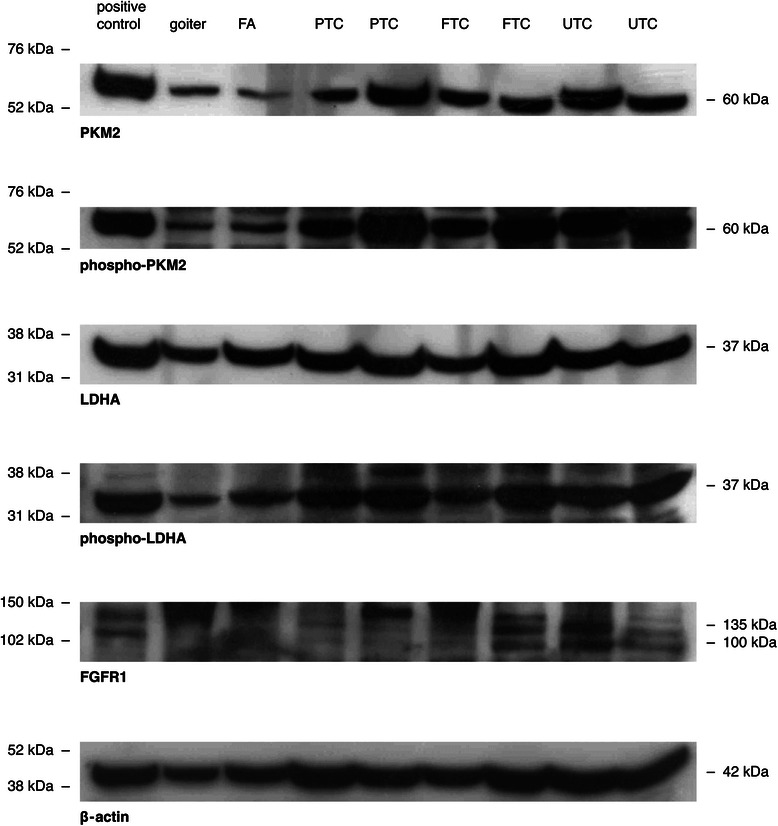
**Protein analysis of PKM2, LDHA and FGFR1 expression in thyroid tissues.** Representative images of western blot performed with total protein lysates obtained from thyroid tissues and antibodies against total-PKM2, Phospho-PKM2, total-LDHA, Phospho-LDHA and FGFR1. Thyroid samples were divided into following histological subgroups: benign goiter (Goiter), follicular adenoma (FA), follicular thyroid cancer (FTC), papillary thyroid cancer (PTC) and undifferentiated thyroid cancer (UTC). ß-actin was used as a normalizing marker.

**Figure 2:**
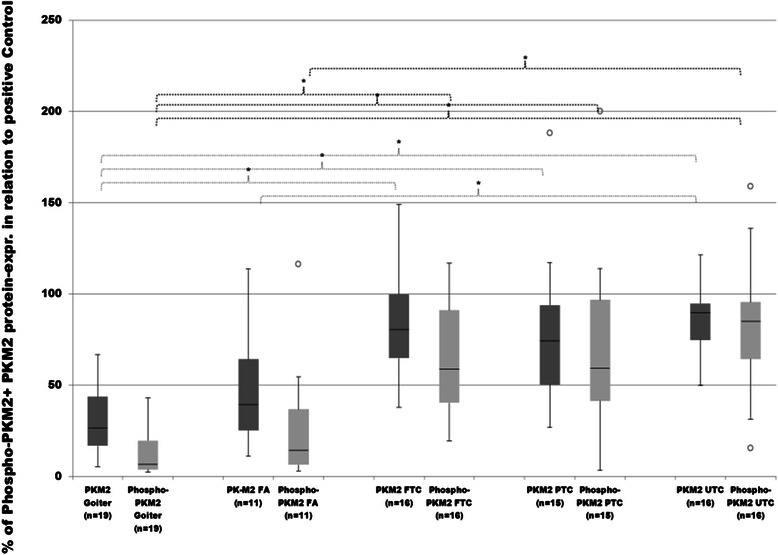
**Evaluation of total- and Phospho-PKM2 protein expression.** Total and y105-phosphorylated PKM2 expressions in goiter, FA, FTC, PTC and UTC were analysed by employment of western blot analysis and evaluated densitometrically with ImageJ software. Protein expression was measured in relation to FTC133 as positive control and then normalized to the expression of ß-actin as normalizing marker. Differences are expressed as percent to positive control defined as 100%. *p < 0.05 indicates a statistical significance.

**Figure 3:**
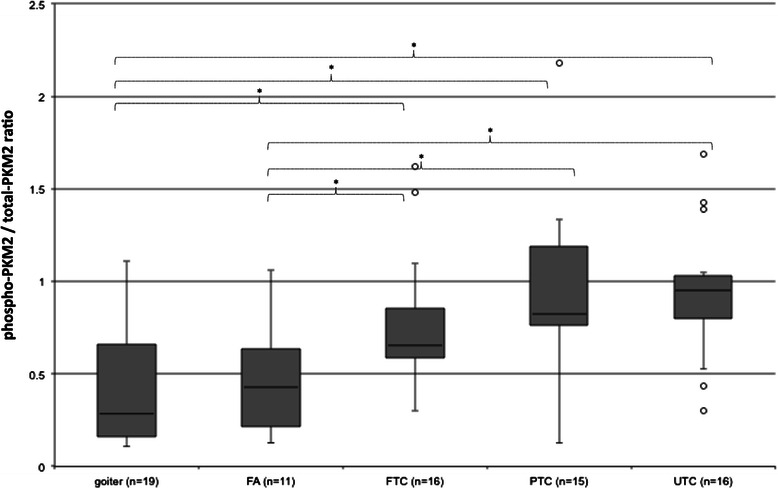
**Phospho-PKM2/total PKM2 ratio.** Total and y105-phosphorylated PKM2 expression in goiter, FA, FTC, PTC and UTC were analysed by employment of western blot (see Figure 1). Phospho-PKM2/total-PKM2 ratio was built to show phospho-PKM2 in relation to total-PKM2. *p < 0.05 indicates a statistical significance.

As demonstrated in Figures 1, 2 and 3, the protein levels of total and phosphorylated PKM2 were expressed noticeably higher in every cancer subgroup as compared to goiter or FA. The highest and significant expression of total- and Phospho-PKM2 was found in UTC as compared to goiter or FA samples. A similar and significantly elevated expression pattern of both PKM2 proteins was also found in other cancer groups. Comparisons between carcinoma tissues and FA revealed a noticeably increased expression of total- and Phospho-PKM2 in FTC, PTC and UTC. However, only the difference between UTC and FA was found significant. Analysis of the median expression of total PKM2 in each histological subgroup revealed the following percentages of expressional values: 26.6% (goiter), 39.3% (FA), 80.5% (FTC), 74.1 % (PTC) and 89.7% (UTC). Median levels of Phospho-PKM2 in each histological subgroup showed 6.4% (goiter), 14.38% (FA), 58.8% (FTC), 59.2% (PTC) and 85% (UTC). The difference between the median of total PKM2 expression (89.7 %) and Phospho-PKM2 levels (85%) was just 4,7% for UTC. This cancer subtype demonstrated the highest relative PKM2 phosphorylation. Phospho-PKM2/total-PKM2 ratio showed significantly increased relative phosphorylation in all cancer subgroups compared to goiter and FA (Figure 3).

**Figure 4:**
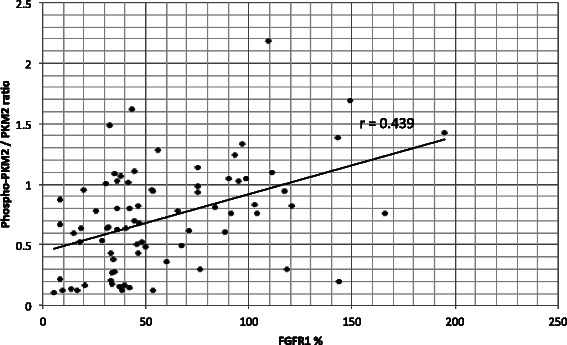
**Correlations between Phospho-PKM2/total-PKM2.** Correlation between ratio of Phospho-PKM2/total-PKM2 and FGFR1 expression. Pearson correlation was applied.

**Figure 5:**
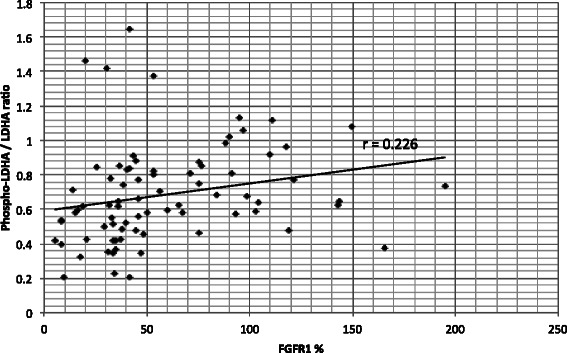
**Correlations between Phospho-LDHA/total-LDHA and FGFR1 expression.** Correlation between ratio of LDHA/total-LDHA and FGFR1 expression. Pearson correlation was applied.

In further analysis, Phospho-PKM2 revealed a significant correlation with FGFR1 expression (r 0.39, p < 0.05) (data not shown). To minimize the confounding factor of increasing PKM2 expression from benign to malignant tissue, a ratio between phosphorylated and total PKM2 expression (Phospho-PKM2/PKM2) was used. This ratio showed an even higher correlation of r 0.439 (p < 0.05) with FGFR1 expression (Figure 4) and also higher as Phospho LDHA/LDHA ratio (Figure 5). FGFR1 and Phospho-PKM2/PKM2 showed a significant correlation of r 0.529, (p < 0.05) in the group of UTC (data not shown).

**Figure 6:**
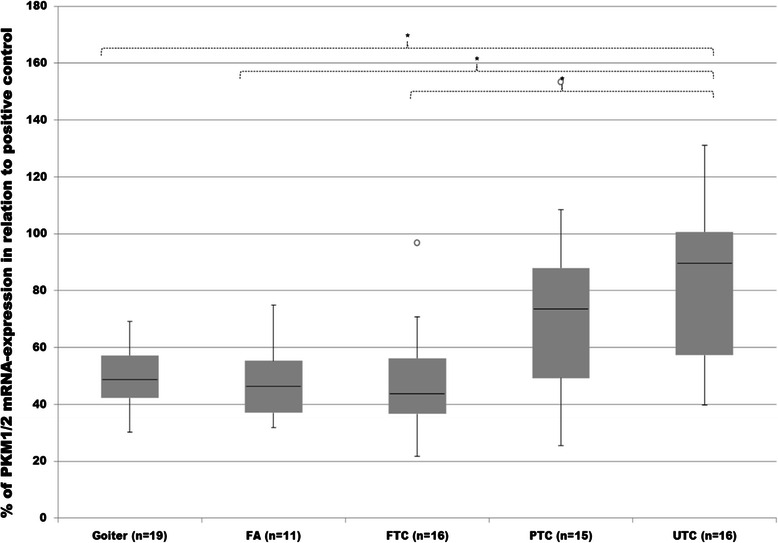
**mRNA expression of Pyruvate Kinase M1 and M2 (PKM1/2).** Expression of PKM1/2 in goiter, FA, FTC, PTC and UTC was analysed by employment of Q-RT-PCR. mRNA expression was measured in relation to FTC133 as positive control and then normalized to the expression of yWHAZ and GAPDH as normalizing markers. Differences are expressed as percent to positive control defined as 100%. *p < 0.05 indicates a statistical significance.

Unfortunately, designing a qPCR primer for analysis including every transcript variant of PKM2 separately without amplification of PKM1 was not possible. This problem was approached by using the primers to amplify PKM1 and PKM2 simultaneously. According to previous studies, PKM1 levels in thyroid tissue are very low as compared to PKM2 [6] (Additional file 1). In this study the focus was on combined PKM1/2 mRNA analysis, which still provided enough information about PKM2 expression. As shown in Figure 6, analysis of mRNA levels revealed significantly higher expression of PKM1/2 in UTC as compared to goiter, FA and FTC. Similar and elevated expression patterns were observed for PTC, however, these differences were not significant.

### Expression and phosphorylation of lactate dehydrogenase A (y 10)

**Figure 7:**
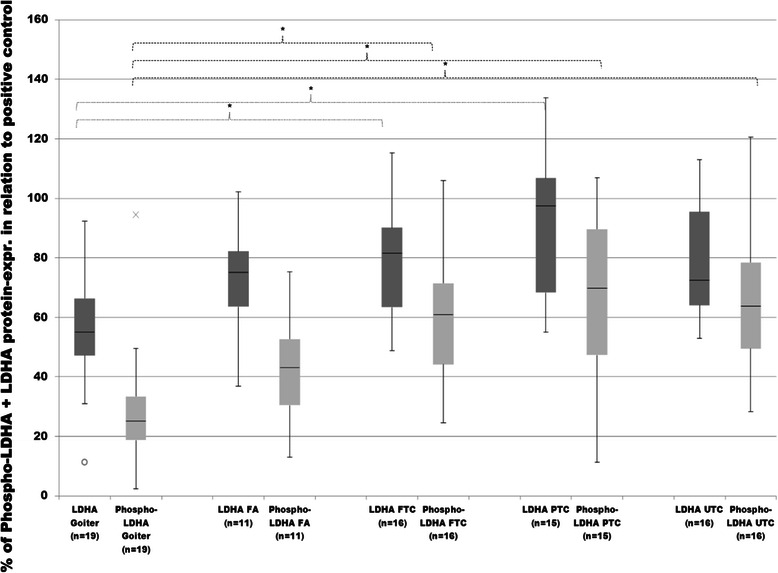
**Evaluation of total- and Phospho-LDHA protein expression.** Total and y10-phosphorylated LDHA expressions in goiter, FA, FTC, PTC and UTC were analysed by employment of western blot analysis and evaluated densitometrically with ImageJ software. Protein expression was measured in relation to FTC133 as positive control and then normalized to the expression of ß-actin as normalizing marker. Differences are expressed as percent to positive control defined as 100%. *p < 0.05 indicates a statistical significance.

**Figure 8:**
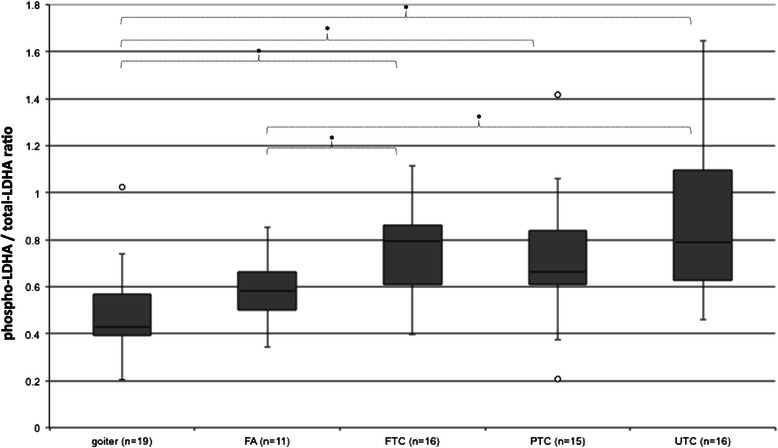
**Phospho-LDHA / total-LDHA ratio.** Total and y10-phosphorylated LDHA expression in goiter, FA, FTC, PTC and UTC were analysed by employment of western blot (see Figure 1). Phospho-PKM2 / total-PKM2 ratio was built to show phosphorylated PKM2 in relation to total PKM2. *p < 0.05 indicates a statistical significance.

In comparison to PKM2, LDHA showed a more stable expression pattern within each group and between the different histological subgroups (Figures 1, 7 and 8). The highest expression of total LDHA was found in PTC. Furthermore, the median expression of PTC was almost twofold higher than in goiter. FTC also revealed significantly higher levels of total LDHAas compared to goiter. UTC and FA demonstrated a noticeable, but not significant increase in total LDHA expression compared to goiter (Figure 7).

Analysis of phosphorylated LDHA revealed higher levels of LDHA in each cancer subgroup (PTC, FTC and UTC) in contrast to goiter. The median expression of total LDHA was 54.9% in goiter, 75% in FA, 81.5% in FTC, 97.4% in PTC and 72.5% in UTC. Median Phospho-LDHA levels were 25.1% in goiter, 43% in FA, 60.8% in FTC, 69.7% in PTC, 63.6% in UTC. PTC showed the highest median expression of total LDHA (97,4%) and the highest median level of Phospho-LDHA (69.7%). UTC revealed a noticeable, however not significant up-regulation of LDHA as compared to goiter. Phospho-LDHA / total LDHA ratio showed significantly increased relative phosphorylation in all cancer subgroups in comparison to goiter (Figure 8). UTC and FTC showed significantly increased Phospho-LDHA/total-LDHA ratio in comparison to follicular adenoma (FA) (Figure 8).

The correlation between the FGFR1 expression and Phospho-LDHA levels was r 0.311 (p < 0.05) (data not shown) and was even lower using a Phospho-LDHA/LDHA ratio; r 0.226 (p < 0.05) (Figure 5). In histological subgroups only FTC showed a significant correlation between FGFR1 and Phospho-LDHA/LDHA ratio at r 0.648 (p < 0.05) (data not shown). All other groups did not show a significant correlation. However, in the group of goiter tissue a correlation of r 0.444 (p = 0.057) (data not shown) was found, being just outside of agreed statistical significance.

**Figure 9:**
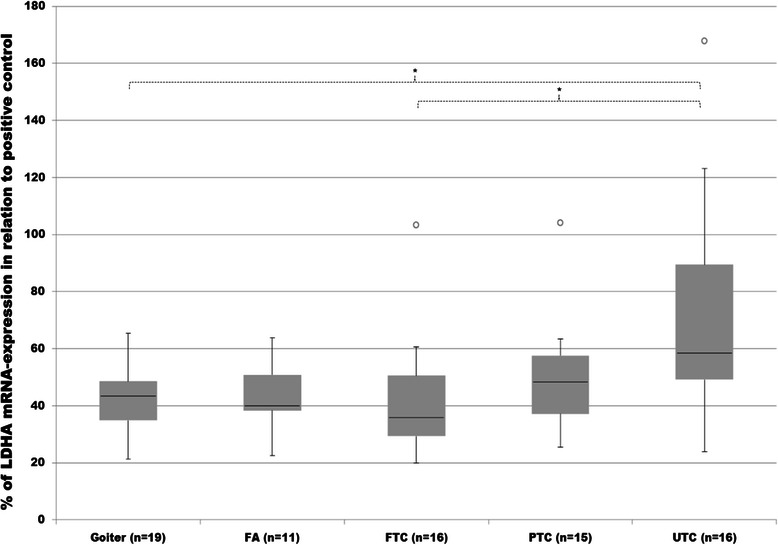
**mRNA expression of Lactate dehydrogenase A (LDHA).** Expression of LDHA in goiter, FA, FTC, PTC and UTC was analysed by employment of Q-RT-PCR analysis. mRNA expression was measured in relation to FTC133 as positive control and then normalized to the expression of yWHAZ and GAPDH as normalizing markers. Differences are expressed as percent to positive control defined as 100%. *p < 0.05 indicates a statistical significance.

**Figure 10:**
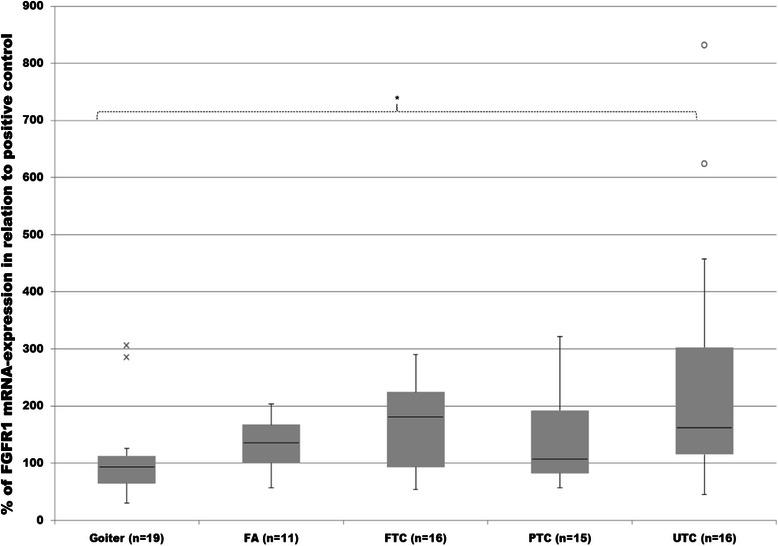
**mRNA expression of Fibroblast growth factor receptor 1 (FGFR1).** Expression of FGFR1 in goiter, FA, FTC, PTC and UTC was analysed by employment of Q-RT-PCR analysis. mRNA expression was measured in relation to FTC133 as positive control and then normalized to the expression of yWHAZ and GAPDH as normalizing markers. Differences are expressed as percent to positive control defined as 100%. *p < 0.05 indicates a statistical significance.

Analysis of LDHA transcripts by employment of RT-PCR analysis revealed significantly up-regulated expression in UTC as compared to goiter. Also the differences between UTC and FTC tissues were significant. Expression of LDHA in PTC showed an increased tendency as compared to goiter or FA, however, these differences were not significant (Figure 9 and 10).

### Expression of fibroblast growth factor receptor 1 (FGFR1)

**Figure 11:**
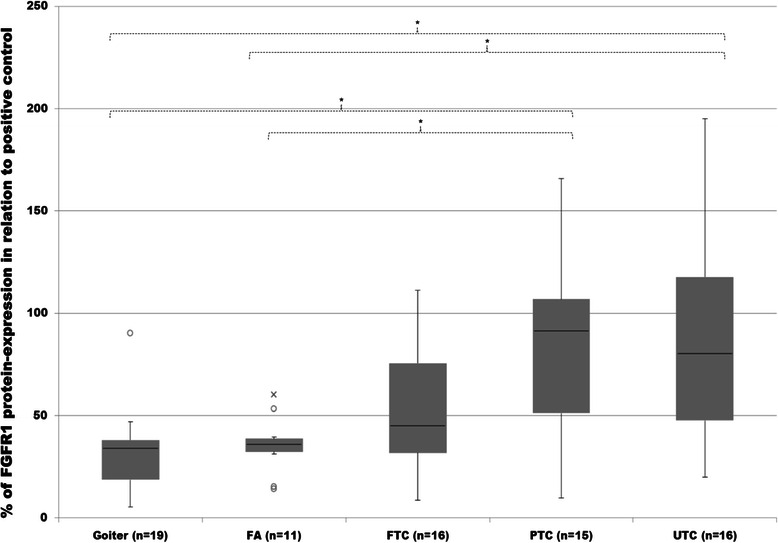
**Evaluation of FGFR1 protein expression.** Expression of FGFR1 in goiter, FA, FTC, PTC and UTC was analysed by employment of western blot analysis and evaluated densitometrically with ImageJ software. Protein expression was measured in relation to FTC133 as positive control and then normalized to the expression of ß-actin as normalizing marker. Differences are expressed in percent to positive control defined as 100%. *p < 0.05 indicates a statistical significance.

FGFR1 protein was expressed in all examined tissue samples. It was up-regulated significantly in PTC and UTC compared to goiter or FA (Figure 11). FTC revealed lower median expression of FGFR1 than in PTC and UTC, however being still noticeably higher than in FA or goiter.

Analysis of FGFR1 mRNA expression revealed a noticeable increase in FA and all cancer subgroups compared to goiter tissue. LDHA was found markedly higher in FTC compared to FA or goiter without a statistical difference observed. Only the differences between UTC and goiter were statistically significant (Figure 9).

### Expression of phosphorylated PKM2 and LDHA under FGFR1 inhibition

**Figure 12:**
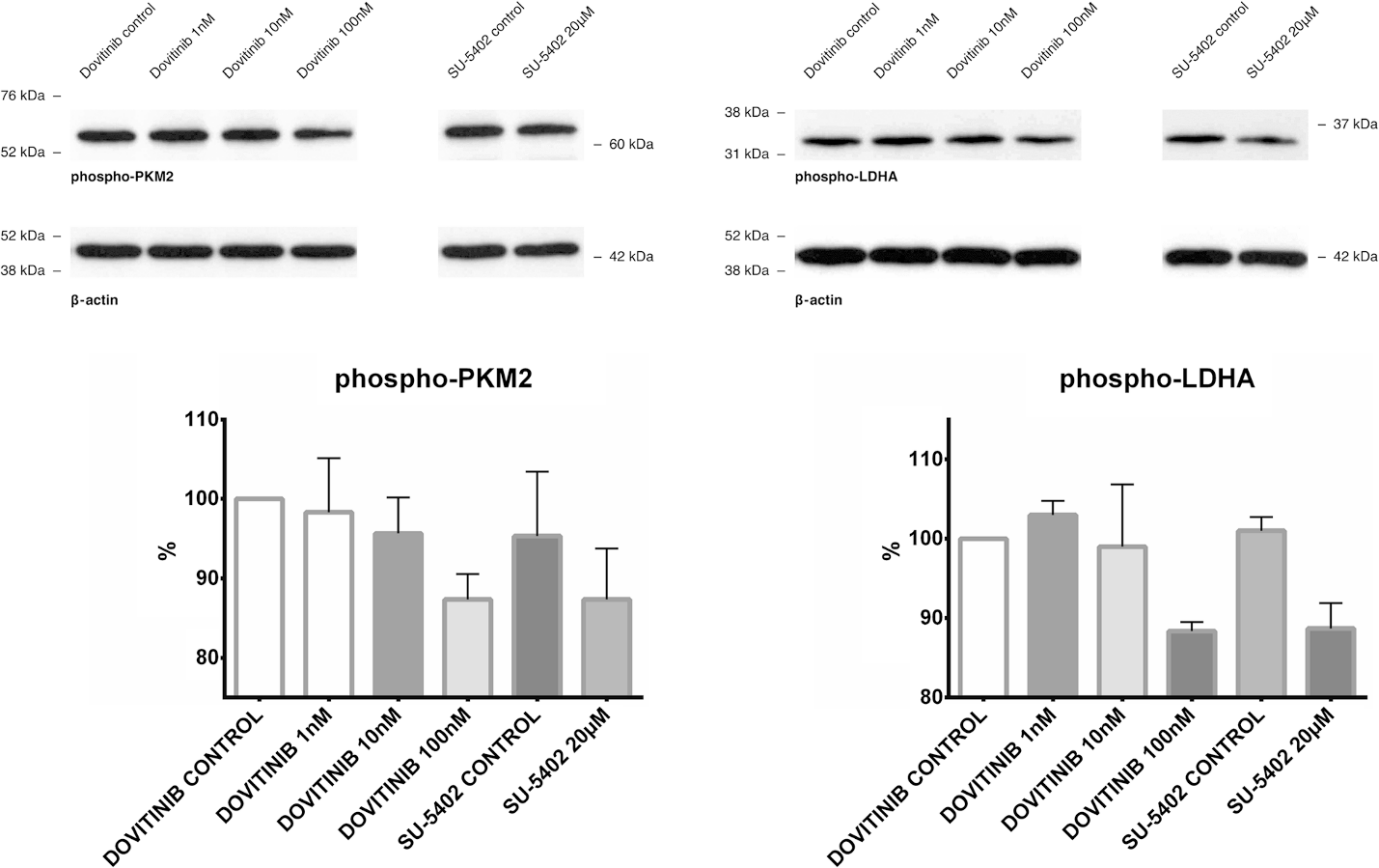
**Inhibition of FGFR and response in phosphorylation status of PKM2 and LDHA.** Inhibiton experiments were performed with Dovitinib and SU-5402, two inhibitors of Fibroblast growth factor receptor (FGFR). Phosphorylation status of PKM2 and LDHA was measured after four hours and showed a significant decrease with Dovitinib 100nM and SU-5402 20 μM for both proteins. DMSO was used as positive control. No significant downregulation of PKM2 and LDHA phosphorylation was detectable with 1nM and 10 nM of Dovitinib.

In order to investigate whether phosphorylation of PKM2 and LDHA is mediated in FGFR1-specific manner, FTC-133 were treated with receptor tyrosine kinase inhibitors Dovitinib and SU-5402. Dovitinib treatment resulted in significant decrease of phosphorylation status at a concentration of 100 nM after four hours of incubation for both PKM2 and LDHA (Figure 12). No significant changes were seen when administered at concentrations of 1 nM and 10 nM. SU-5402 administration led to a sigificant decrease of PKM2 and LDHA phosphorylation at a concentration of 20 μM (Figure 12).

## Discussion

In this study we demonstrated that total and phosphorylated PKM2 and LDHA proteins are significantly up-regulated in thyroid cancer tissues as compared to goiter. To our knowledge, in addition to previous PKM2 results in thyroid cancer [6], this is the first report demonstrating an increased phosphorylation status of both proteins in thyroid cancer.

It is well known that in addition to the Warburg effect, expression of PKM2 and LDHA in tumour tissues may be regulated by Hypoxia inducible factor 1-alpha (HIF1a) and c-Myc. Both oncogenes were also reported to be elevated in thyroid malignancies [31-36]. With regards to our data, we found that in thyroid carcinoma tissues PKM2 is expressed more abundantly than LDHA. Based on these observations we suggest that the Warburg effect is not the only factor affecting PKM2 regulation in thyroid cancer, especially in UTC. Furthermore, as reported in our study, different expressional changes observed between PKM2 and LDHA may result from other than the enzymatic role of PKM2 in gene transcription and proliferation [16,37,38].

Various other studies had previously demonstrated increased levels of LDHA and PKM2 in tumour tissues and their important role in proliferation and survival of malignant cells [7,23,39]. In accordance with these findings we were able to show that thyroid carcinoma samples revealed not only significantly elevated levels of total PKM2 and LDHA compared to goiter or FA, but this expressional increase correlated directly with phosphorylated forms of both proteins. Furthermore, our data suggests that in patients with thyreoglobulin-negative thyroid cancer, phosphorylated PKM2 and LDHA may represent a more valuable diagnostic potential than total expressions of these proteins. Indeed, we found that the FGFR1 expression correlated well with phosphorylation status of both proteins. These results suggest a possible, but not only FGFR1-mediated phosphorylation mechanism of PKM2 and LDHA in thyroid carcinoma. However, increased phosphorylation of various proteins and up-regulation of FGFR are known to occur in cancer, being a possible confounder of our conclusion.

We therefore conducted additional cell culture experiments and were able to demonstrate that phosphorylation of PKM2 and LDHA occurs in an FGFR1-specific manner. Inhibition of FGFR1 in the thyroid cancer cell line FTC133 resulted in significantly decreased phosphorylation status of both investigated enzymes. It is worth noting that tyrosine kinase inhibitors like Dovitinib or SU-5402 could be a therapeutic option to target the Warburg effect in thyroid cancer cells.

Discrimination between follicular adenoma (FA) and FTC is often a challenge. Based on our data we noticed a significantly increased relative phosphorylation of PKM2 and LDHA (Figures 3 and 8) in FTC in comparison to FA. However, studies with early stage follicular thyroid cancer are necessary to determine whether relative phosphorylation could be a tool to discriminate FTC from FA.

## Conclusion

In summary, we demonstrated that increased levels of total and phosphorylated forms of PKM2 and LDHA in malignant tissues represent a novel expressional signature with diagnostic potential for thyroid cancer.

## Additional files

Additional file 1: **Representative images of western blot performed with total protein lysates obtained from thyroid tissues and antibodies against PKM2 and total PKM (Pyruvate Kinase M2 and M1).** 20 thyroid samples were analyzed to show correlation between PKM2 and total PKM. The Pearson correlation coefficient was 0.95, concluding that PKM2 is by far the predominant isoform in all examined thyroid tissue.
Additional file 2: **Western blot images performed with total protein lysates from three thyroid cancer cell lines: FTC133 (follicular thyroid cancer cell line), 8505C (undifferentiated thyroid cancer cell line) and B-CPAP (papillary thyroid cancer cell line).** They were stained with antibodies against PKM1, PKM2 total PKM and ß-actin and showed stable expression of all examined proteins. Subsequently, FTC133 was chosen as positive control.
