# Supplementary figures and images for: Phosphorylation of pyruvate kinase M2 and lactate dehydrogenase A by fibroblast growth factor receptor 1 in benign and malignant thyroid tissue
Source: BMC Cancer. 2015 Mar 18;15:140. doi: 10.1186/s12885-015-1135-y (PMC4393606; doi:10.1186/s12885-015-1135-y)

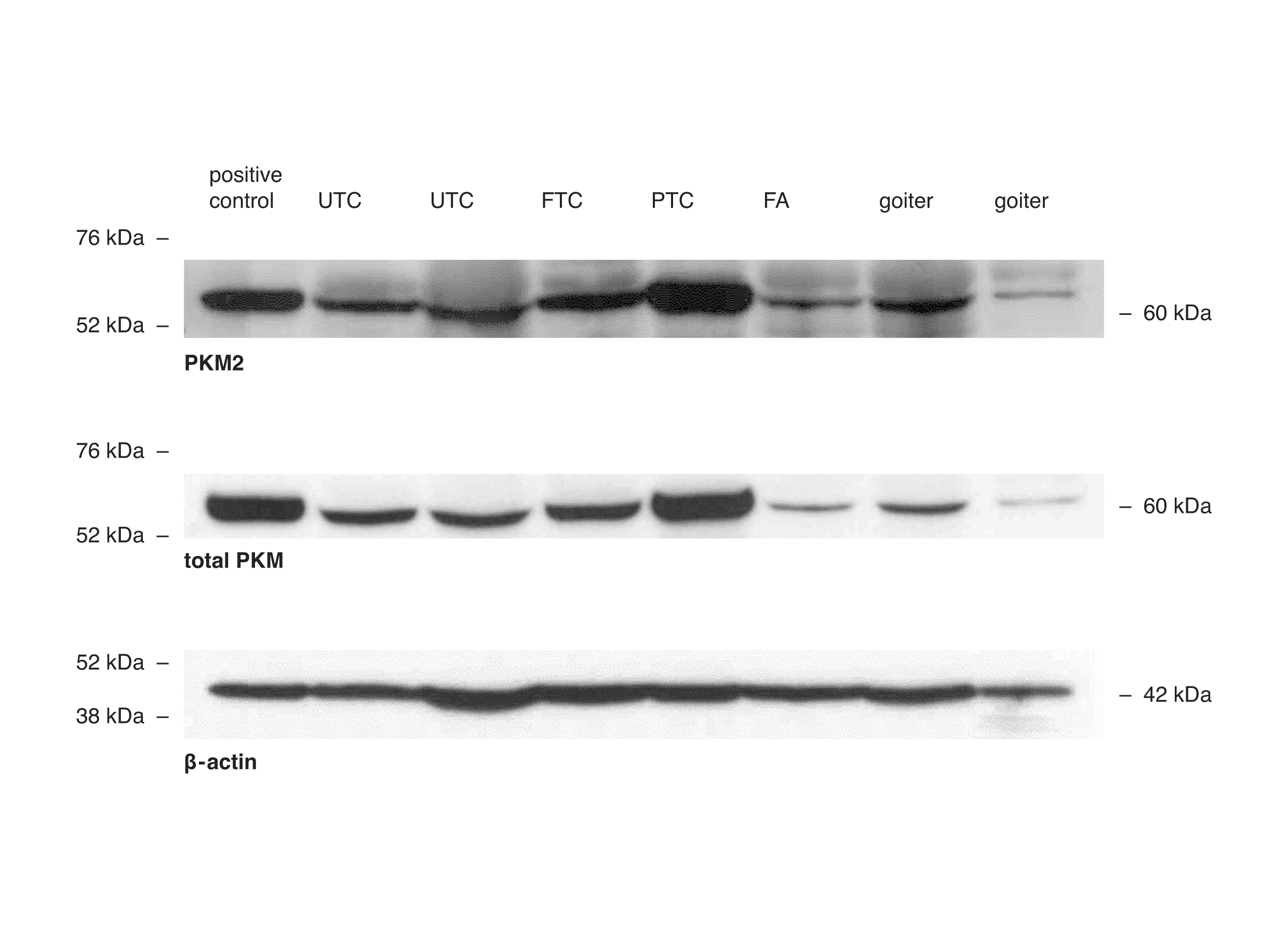

Supplement: Additional file 1: — Representative images of western blot performed with total protein lysates obtained from thyroid tissues and antibodies against PKM2 and total PKM (Pyruvate Kinase M2 and M1). 20 thyroid samples were analyzed to show correlation between PKM2 and total PKM. The Pearson correlation coefficient was 0.95, concluding that PKM2 is by far the predominant isoform in all examined thyroid tissue. [file 12885_2015_1135_MOESM1_ESM.tiff]

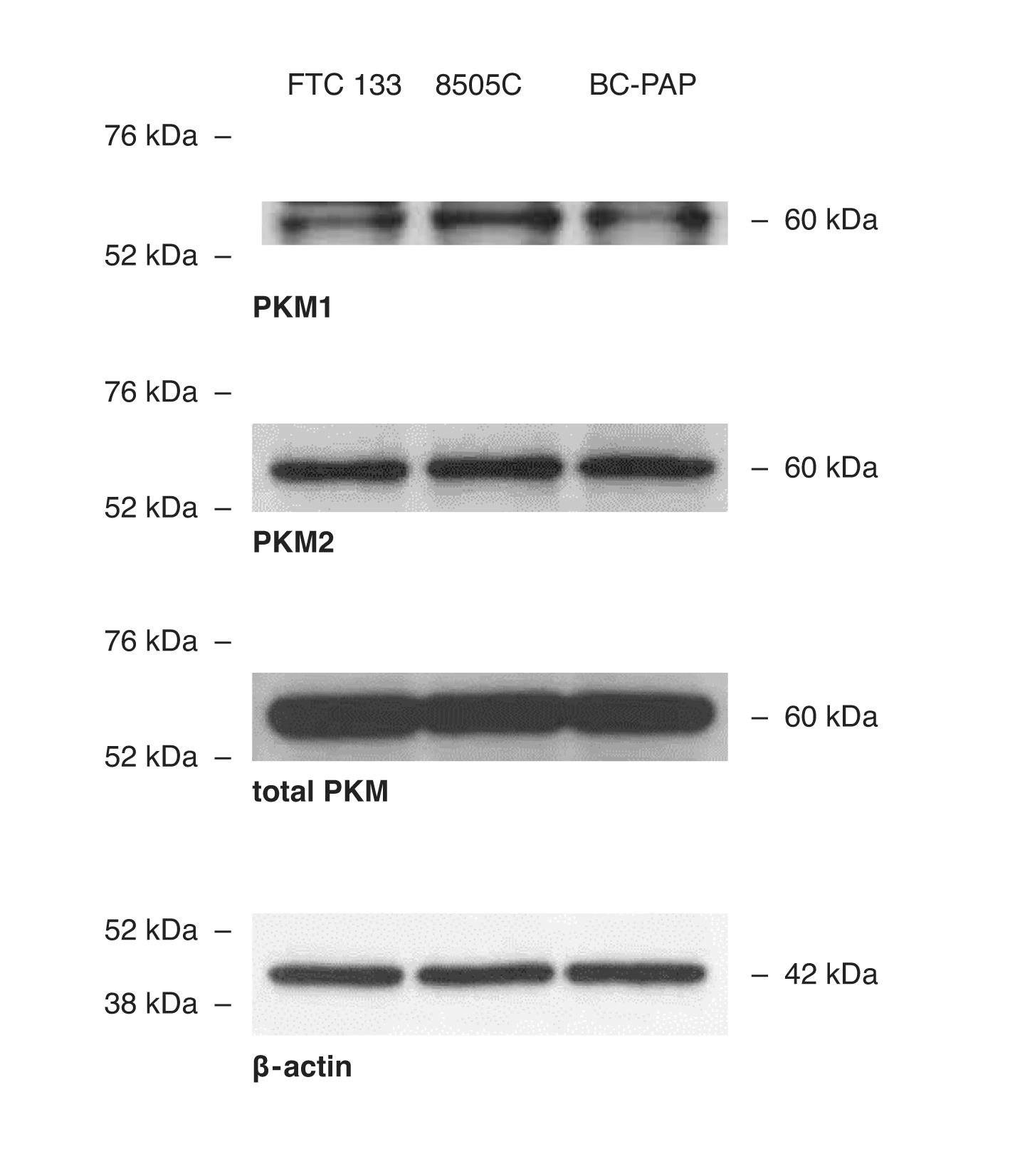

Supplement: Additional file 2: — Western blot images performed with total protein lysates from three thyroid cancer cell lines: FTC133 (follicular thyroid cancer cell line), 8505C (undifferentiated thyroid cancer cell line) and B-CPAP (papillary thyroid cancer cell line). They were stained with antibodies against PKM1, PKM2 total PKM and ß-actin and showed stable expression of all examined proteins. Subsequently, FTC133 was chosen as positive control. [file 12885_2015_1135_MOESM2_ESM.tiff]
